# Antioxidants prevent inflammation and preserve the optic projection and visual function in experimental neurotrauma

**DOI:** 10.1038/s41419-018-1061-4

**Published:** 2018-10-26

**Authors:** Alexandra Bernardo-Colón, Victoria Vest, Adrienne Clark, Melissa L. Cooper, David J. Calkins, Fiona E. Harrison, Tonia S. Rex

**Affiliations:** 1 Department of Ophthalmology and Visual Sciences, Vanderbilt Eye Institute, Vanderbilt University Medical Center, Nashville, TN 37232 USA; 2 Department of Medicine, Vanderbilt University Medical Center, Nashville, TN 37232 USA

## Abstract

We investigated the role of oxidative stress and the inflammasome in trauma-induced axon degeneration and vision loss using a mouse model. The left eyes of male mice were exposed to over-pressure air waves. Wild-type C57Bl/6 mice were fed normal, high-vitamin-E (VitE), ketogenic or ketogenic-control diets. Mice lacking the ability to produce vitamin C (VitC) were maintained on a low-VitC diet. Visual evoked potentials (VEPs) and retinal superoxide levels were measured in vivo. Tissue was collected for biochemical and histological analysis. Injury increased retinal superoxide, decreased SOD2, and increased cleaved caspase-1, IL-1α, IL-1β, and IL-18 levels. Low-VitC exacerbated the changes and the high-VitE diet mitigated them, suggesting that oxidative stress led to the increase in IL-1α and activation of the inflammasome. The injury caused loss of nearly 50% of optic nerve axons at 2 weeks and astrocyte hypertrophy in mice on normal diet, both of which were prevented by the high-VitE diet. The VEP amplitude was decreased after injury in both control-diet and low-VitC mice, but not in the high-VitE-diet mice. The ketogenic diet also prevented the increase in superoxide levels and IL-1α, but had no effect on IL-1β. Despite this, the ketogenic diet preserved optic nerve axons, prevented astrocyte hypertrophy, and preserved the VEP amplitude. These data suggest that oxidative stress induces priming and activation of the inflammasome pathway after neurotrauma of the visual system. Further, blocking the activation of the inflammasome pathway may be an effective post-injury intervention.

## Introduction

Central nervous system (CNS) trauma can lead to secondary neurodegeneration and worsening functional impairments. Here we explore CNS trauma in the context of the visual system. The retina and optic nerve (ON) are accessible regions of the CNS, allowing for direct visualization and delivery of agents. The location of retinal ganglion cells (RGCs) and their axons in two different spatial regions allows for study of each separately. Finally, the axons in the ON are unidirectional, making study of axon transport and damage simpler. Herein we present our findings of the mechanisms underlying secondary ON degeneration after closed-globe eye injury.

Damage to the retina and ON (traumatic optic neuropathy, TON) is associated with worse visual outcomes^1,2^. The incidence of TON in US traumatic brain injury (TBI) patients range from 0.5 to 5%^3,4^. The incidence of TON in the military may be as high as 15%, but rates are confounded by referral bias, additional injuries, and limited information^1^. Nearly 2/3 of the service members with eye injuries also have TBI^5^. Approximately 10% of TON patients develop vision loss with ON pallor weeks after injury, providing an opportunity for medical intervention^6^. High-dose steroids are sometimes prescribed; however, studies have failed to demonstrate benefit over observation alone^7^.

Our model of ocular blast trauma damages the retina and ON while avoiding potentially confounding damage to visual pathways in the brain^8,9^. A single 26-psi air blast induced regions of cell death and slight axon degeneration in the ON at 1 month after injury^8^. Since most military and some civilian (i.e. sports) TBI are due to repeat trauma^10^, we generated a paradigm of repeat air-blast injury. We show that repeat exposure to a 15-psi air blast results in faster and more extensive axon degeneration.

The earliest molecular events in the retina after a single air blast are increased nitrotyrosine and caspase-1. Nitrotyrosine is a marker for peroxynitrite, which is formed from superoxide and nitric oxide, and caspase-1 is required for inflammasome pathway activation (for a review see ref. ^11^)^8^. Increased labeling was initially localized to small regions of the inner retina but by 1 month it spanned the entire inner retina. The inflammasome pathway is primed through cell surface receptors including the IL-1R, which induce expression of inflammasome proteins, pro-IL-1β, and pro-IL-18. A second signal activates the pathway, inducing inflammasome complex formation and cleavage of pro-caspase-1. Caspase-1 cleaves pro-IL-1β and pro-IL-18 into their active forms and they amplify the pathway, causing a vicious cycle that can ultimately lead to pyroptotic cell death^12^. IL-1α is considered an “alarmin” because it can bind to the IL-1R in its pro- or cleaved form, initiating the inflammasome pathway^13^. We hypothesized that trauma-induced reactive oxygen species (ROS) activate the inflammasome and is responsible for secondary axon degeneration and vision loss after trauma. To test this we used diets to alter the antioxidant capacity of the retina. Our results show that ROS play a critical role in secondary axon degeneration and that the damage is mediated, in part, by activation of the inflammasome pathway.

## Results

### Repeat- and single-blast injury activate the same molecular pathways

**Fig. 1:**
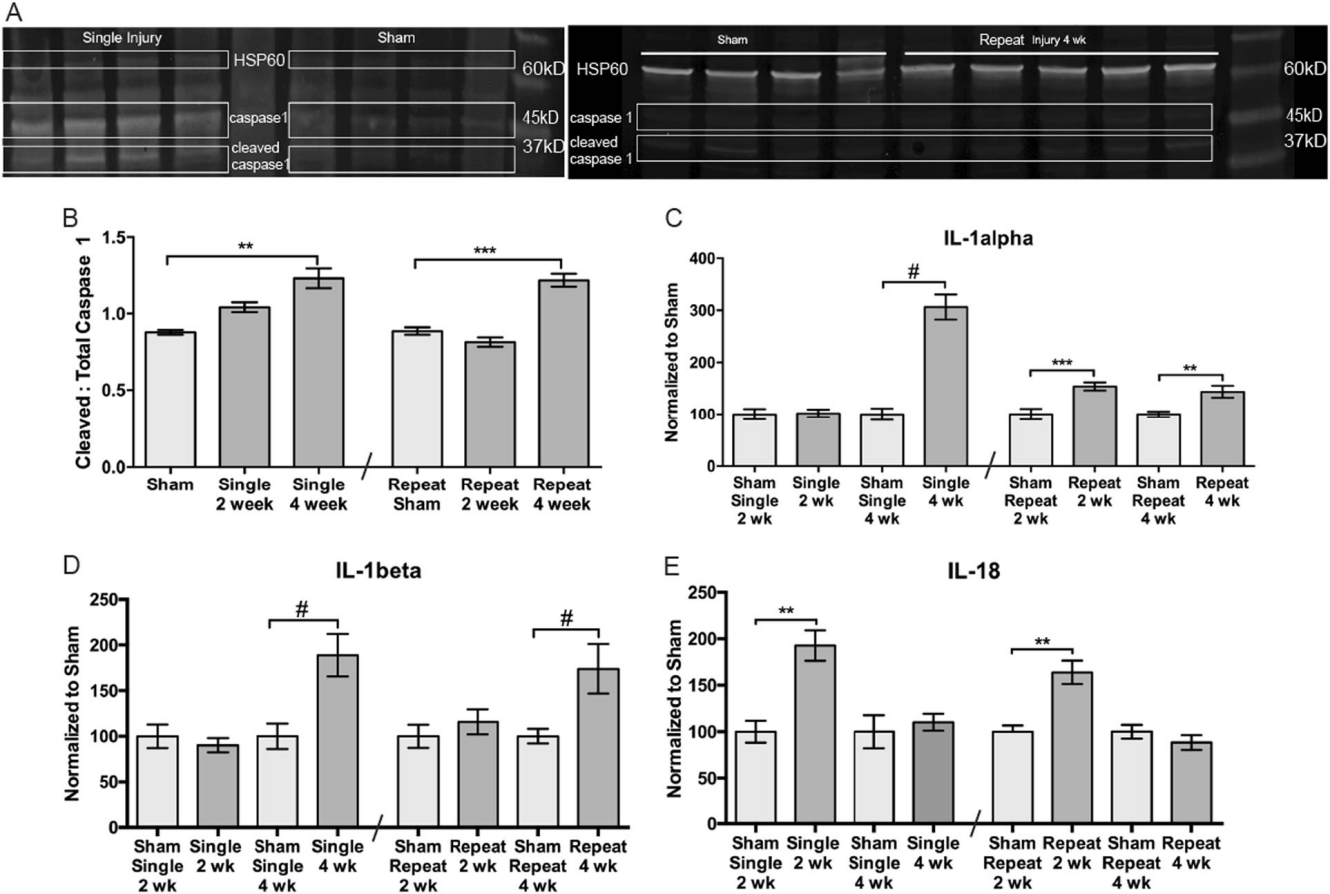
The inflammasome pathway is activated in the retina after single or repeat blast injury to the eye. **a** Western blot of caspase-1 in sham, single injury, and repeat injury mice at 4 weeks after injury. **b** Quantification of cleaved to total caspase-1 showing an increase at 4 weeks in both single and repeat injury groups. **c** Quantification of IL-1α after single or repeat blast. IL-1α is increased at 4 weeks after a single blast and at both 2 and 4 weeks after a repeat blast. **d** Quantification of IL-1β after single or repeat blast showing an increase at 4 weeks after either injury. **e** Quantification of IL-18 after single or repeat blast showing a transient increase at 2 weeks after injury for both groups. This experiment was repeated twice. *n* = 5 for all groups. **p < 0.01, ***p < 0.001, ^#^*p* < 0.0001

We quantified cleaved:total caspase-1 to quantify activation (Fig. 1a, b). The levels were comparable to sham at 2 weeks post injury (Fig. 1b). The ratio was increased by 40% in the single- and 37% in the repeat-blast retinas at 4 weeks post injury as compared to shams (Fig. 1b). Levels of the inflammasomes, NLRP1, and NLRP3 were unchanged from shams (data not shown). Since these proteins could still be active without their levels being increased, this does not rule out their involvement^11^. We performed a 24-plex cytokine ELISA, and for both blast groups we only detected increases in IL-1α and IL-1β (Fig. 1c, d). The levels of IL-1α were unchanged from shams at 2 weeks, but increased 138% at 4 weeks after single injury (Fig. 1c). After repeat injury, IL-1α levels were elevated at both 2 and 4 weeks by 55% and 72%, respectively, above shams (Fig. 1c). The levels of IL-1β were unchanged from shams at 2 weeks after either injury (Fig. 1d). However, 4 weeks after either injury, IL-1β levels increased 51% and 106%, respectively, above shams (Fig. 1d). Since IL-18 was not represented on the multiplex ELISA, we assessed it separately (Fig. 1e). IL-18 levels increased 78% and 56%, respectively, above shams at 2 weeks after either single or repeat injury and returned to sham levels at 4 weeks (Fig. 1j). We also measured levels of thioredoxin interacting protein (TXNIP), a protein that activates the inflammasome in response to ROS. Its levels were unchanged after injury (data not shown); however, this does not exclude the possibility of increased activation by phosphorylation.

### ROS is a primary driver of secondary axon degeneration after ocular trauma

**Fig. 2:**
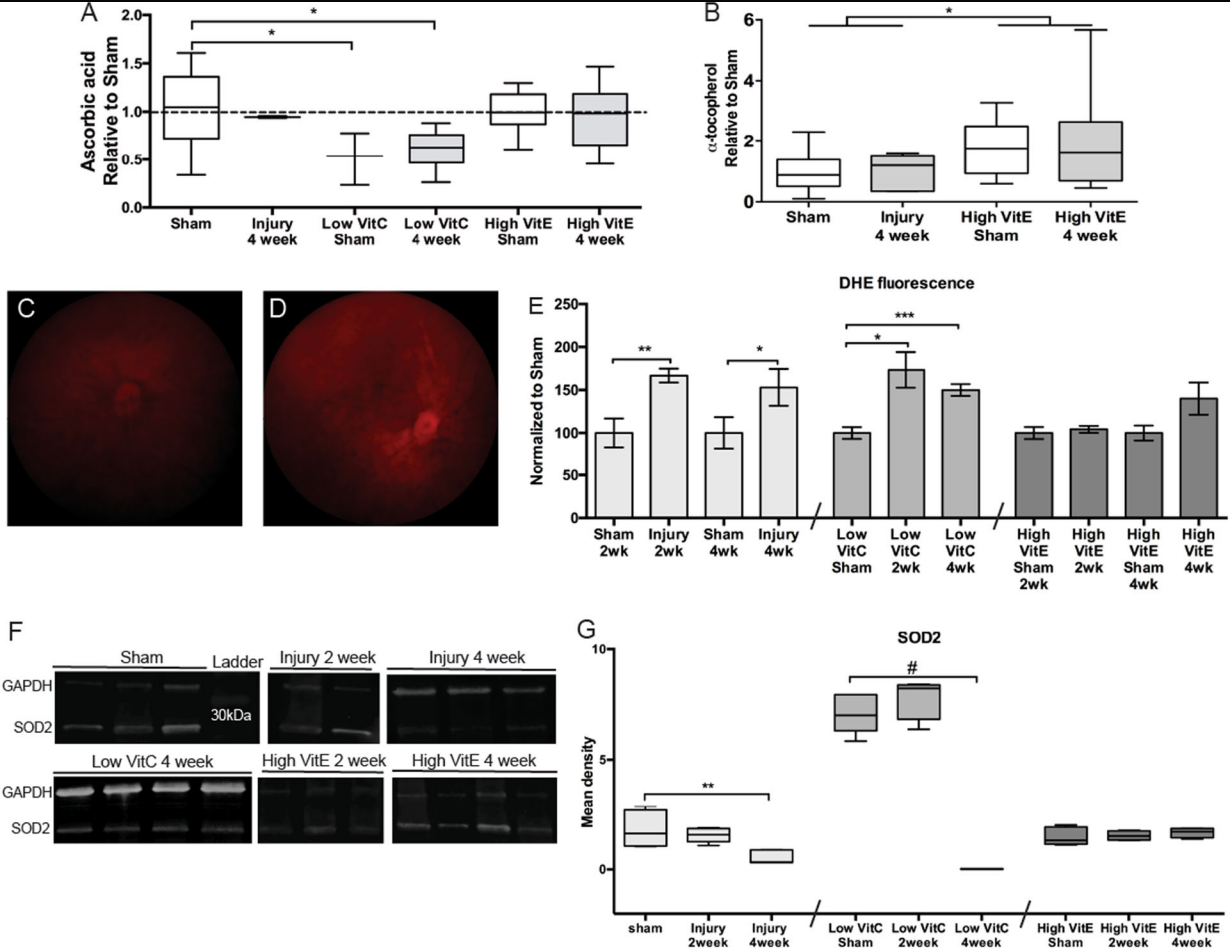
Diets alter tissue levels of VitC, VitE, and superoxide after blast. **a** Quantification of retina ascorbic acid (VitC) levels. Retinas of Gulo^-/-^ mice provided a low-VitC content diet had less ascorbic acid, *p < 0.05. Retinas of wild-type mice fed a high-VitE diet contained normal levels of ascorbic acid. **b** Quantification of retina α-tocopherol (VitE) levels. Retinas of wild-type mice fed a high-VitE diet contained higher levels of α-tocopherol. **c**, **d** Representative images of DHE fluorescence (superoxide levels) in the retinas of sham (**c**) and repeat blast-exposed (**d**) mice. **e** Quantification of retina DHE fluorescence in normal, low-VitC, and high-VitE-diet mice. Levels are increased in the 2 and 4 weeks after blast injury in both the control and low-VitC mice, but not in the high-VitE mice. **f** Western blots of SOD2 levels in retinas from sham, and 2- or 4-week post-blast mice on control, low-VitC, or high-VitE diets. **g** Quantification of SOD2 levels. SOD2 levels are decreased at 4 weeks after injury in both normal-diet and low-VitC-diet mice, but not the high-VitE mice. Low VitC retinas contained increased levels of SOD2 in sham and 2 week post-blast mice, suggesting an endogenous compensatory effect of the low-VitC diet. This experiment was repeated twice. *n* = 5 for all groups. *p < 0.05, **p < 0.01, ***p < 0.001, ^#^*p* < 0.0001

We manipulated levels of retinal vitamin C (VitC) and vitamin E (VitE) in order to examine the contribution of ROS to trauma-induced inflammasome activation, neuronal degeneration, and vision loss. The low-VitC diet provides sufficient levels to avoid scurvy, but does not saturate the sodium-dependent VitC transporter, type 2 at the blood–brain barrier^14^. This dose may also be clinically relevant since significantly depleted or frankly deficient serum VitC levels (< 28 μM) are observed in up to 50% of otherwise healthy populations (for a review see^15^). Deficiency is greater in smokers^16,17^ and persistent hypovitaminosis for VitC is observed in veterans^18^. Blast had no effect on endogenous retinal VitC levels (Fig. 2a). We detected a 50% and 40% decrease in retinal VitC levels in sham and blast low-VitC mice, respectively, as compared to controls (Fig. 2a). The retinas of high-VitE-diet mice contained comparable levels of VitC as controls (Fig. 2a). This was expected because wild-type mice can moderate VitC synthesis according to the requirement. VitC in these diets is provided in excess to ensure sufficient VitC is available to recycle oxidized VitE from its radical form^19^. The retinas of high-VitE mice contained 70% (sham) and 90% (blast-injured) more α-tocopherol (VitE) than control mice, *p* < 0.05 (Fig. 2b). Blast had no effect on VitE content (Fig. 2b).

Blast caused an increase in dihydroethidium (DHE) fluorescence, a marker of superoxide, as compared to shams (Fig. 2c, d). An increase of over 50% was detected at 2 and 4 weeks after repeat blast in control-diet mice (Fig. 2e). A similar increase was detected in retinas from low-VitC-diet mice (Fig. 2e). The lack of additional increase suggests that we may have reached the technical maximum of the assay. In contrast, fluorescence remained at sham levels in the retinas of high-VitE-diet mice at both 2 and 4 weeks after blast (Fig. 2e). Quantification by quadrant did not yield a regional effect (data not shown). Since the primary source of superoxide is the mitochondria, we measured levels of the mitochondrial superoxide dismutase (MnSOD/SOD2) (Fig. 2f). SOD2 levels were decreased by 72% at 4, but not 2 weeks after injury (Fig. 2g). Low-VitC diet mice exhibited a 286% increase over control shams regardless of injury (Fig. 2g). However, they exhibited a decrease in SOD2 at 4 weeks after blast to almost undetectable levels (Fig. 2g). Treatment with high VitE prevented the decrease in SOD2 after blast (Fig. 2g).

**Fig. 3:**
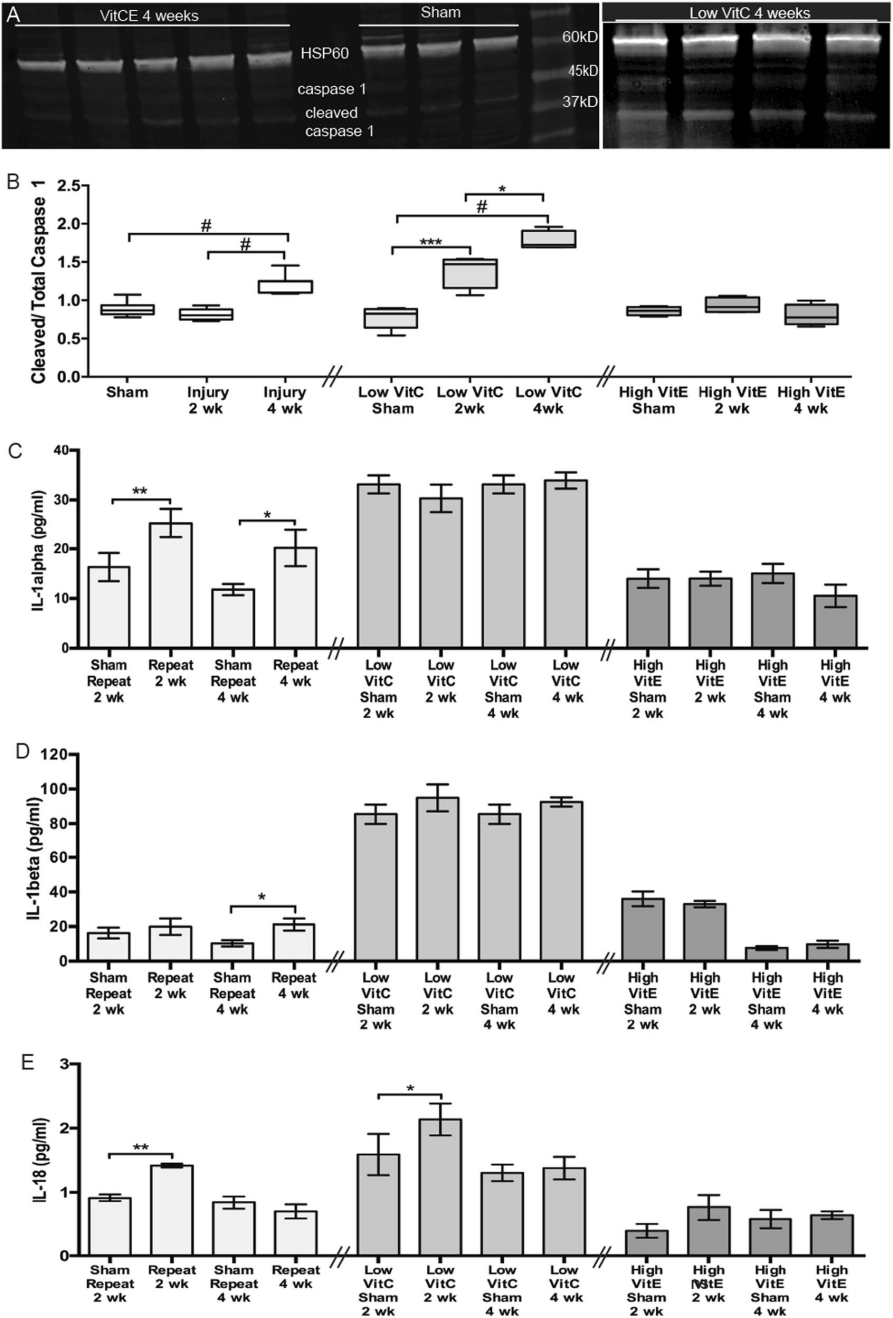
Diets alter activation of the inflammasome pathway after injury. **a** Western blots of cleaved and total caspase-1 in sham and post-blast mice treated with a high-VitE or low-VitC diet. **b** Quantification of cleaved to total caspase-1 shows a greater increase in low-VitC retinas than control-diet mice. There was no increase in activated caspase -1 in the retinas of mice on a high-VitE diet. **c**, **d** Quantification of IL-1α (**c**) and IL-1β (**d**) shows that levels are elevated in retinas of mice on a low-VitC diet regardless of injury. There was no increase in IL-1α or IL-1β levels in the retinas of high-VitE-diet mice. **e** Quantification of IL-18 levels also shows an overall elevation in retinas from mice on a low-VitC diet. In addition, these mice also had a transient increase at 2 weeks after injury as in control-diet mice. There was no increase in IL-18 in the retinas from the high-VitE-diet mice. This experiment was repeated twice. *n* = 5 for all groups. *p < 0.05, **p < 0.01, ***p < 0.001, ^#^*p* < 0.0001

In the low-VitC mice, the decreased antioxidant capacity resulted in a 79% and 129% increase in the levels of cleaved:total caspase-1 at 2 and 4 weeks after blast, respectively, as compared to the low-VitC shams (Fig. 3a, b). This is an earlier and greater increase than in wild-type mice (Fig. 1b, 3b). The retinas of low-VitC-diet mice also exhibited a 97% increase in IL-1α levels as compared to wild-type mice regardless of injury, demonstrating a higher baseline inflammatory state (Fig. 3c). No further increase was detected after blast (Fig. 3c). Similarly, the levels of IL-1β in the low VitC retinas were elevated as compared to sham retinas regardless of injury and no additional increase was detected after blast (Fig. 3d). IL-18 baseline levels were also increased in the retinas of low-VitC-diet as compared to sham levels in retinas of control-diet mice (Fig. 3e). In addition, there was a further increase in IL-18 levels at 2, but not 4 weeks after blast as compared to shams (Fig. 3e). In contrast, high-VitE-diet mice maintained levels of cleaved:total caspase-1, IL-18, IL-1α, and IL-1β at sham levels after blast (Fig. 3a-e).

**Fig. 4:**
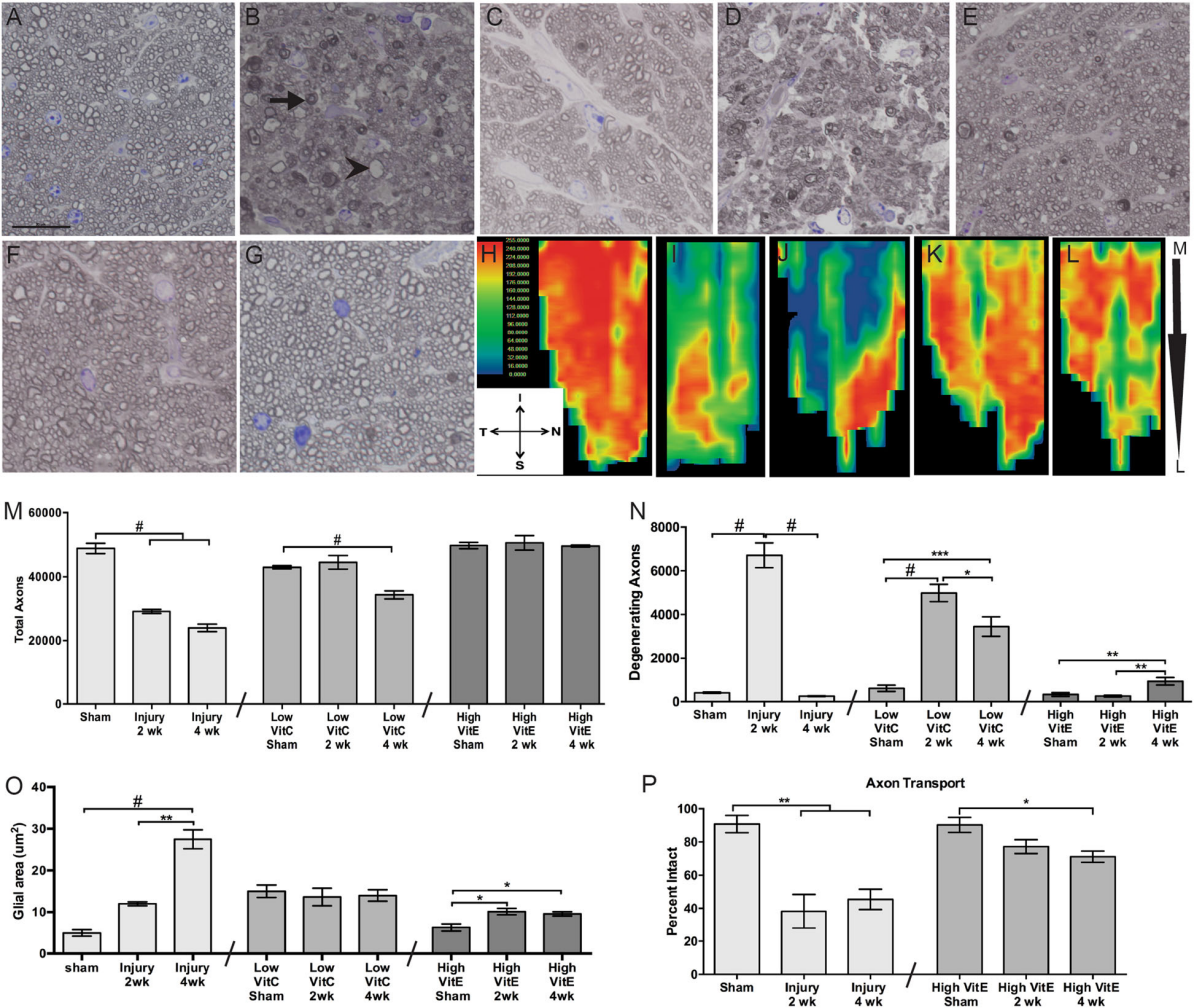
High-VitE diet prevents injury-induced axon degeneration and axon transport deficits. **a**-**g** Representative brightfield micrographs of ON from **a** sham (scale represents 20 μm and applies to all micrographs); **b** 2-week post-blast (arrow indicates re-myelination; arrowhead indicates Wallerian degeneration); **c** 4 week post-blast (note the loss of small axons); **d** 2 week post-blast low-VitC-diet; **e** 4 week post-blast low-VitC-diet; **f** 2-week post-blast high-VitE-diet; and **g** 4 week post-blast high-VitE-diet mice. **h**-**l** Representative heat maps of fluorescently tagged CTB in the superior colliculus of sham (**h**), 2 week post-blast (**i**), 4 week post-blast (**j**), 2 week post-blast high-VitE-diet (**k**), and 4 week post-blast high-VitE-diet (**l**) mice. Medial (M), lateral superior colliculs (L), inferior (I), nasal (N), superior (S), and temporal (T) regions of the retinotopic map on the superior colliculus. **m** Quantification of total axons at 2 and 4 weeks after blast exposure in all diet groups as compared to their respective shams. Total axons were preserved in the high-VitE-diet group only. **n** Quantification of degenerative axons at 2 and 4 weeks after blast exposure in all diet groups as compared to their respective shams. Axon degeneration was more sustained in the low-VitC-diet group and was lowest overall in the high-VitE-diet group. **o** Quantification of cross-sectional ON glial area at 2 and 4 weeks after blast exposure in all diet groups as compared to their respective shams. Baseline glial area was elevated in the low-VitC-diet group and was unchanged after injury. The increase in glia area was less than in controls in the high-VitE-diet group. **p** Quantification of axon transport based on CTB fluorescence levels in the superior colliculus. Axon transport was preserved in the mice on the high-VitE diet. This experiment was repeated twice. *n* = 5 for all groups. *p < 0.05, **p < 0.01, ***p < 0.001, ^#^*p* < 0.0001

The ON of sham mice contained axons with condensed myelin and clear axoplasms along with glial cells with thin processes (Fig. 4a). At 2 weeks after injury many different degenerative profiles were evident, including vacuolization (arrows) and hyper-myelination (arrowheads) (Fig. 4b). At 4 weeks after injury, there were still many degenerative profiles present in the ON and many small axons were missing (Fig. 4c). The ON from low-VitC mice exhibited glial hypertrophy in addition to degenerating profiles at both time points (Fig. 4d, e). In contrast, the ON from high-VitE mice contained many fewer degenerating axons and the glial morphology appeared more similar to that of shams at 2 weeks post injury (Fig. 4f). At 4 weeks post injury, there were more axon profiles and smaller glia than in the control diet of 2 week post-injury ON (Fig. 4g). However, some axon degeneration was detected in the high-VitE-treated mice at 4 weeks after injury (Fig. 4g). Axon transport was measured by quantifying fluorescence in the superior colliculus after intravitreal injection of fluorescently labeled cholera toxin B (CTB; Fig. 4h-l). Transport loss was apparent at 2 weeks after injury, with a greater loss in the medial region of the superior colliculus (Fig. 4i). The same pattern of loss, but with greater deficit, was detected at 4 weeks post injury (Fig. 4j). Much of the transport loss was prevented with the high-VitE diet, with the exception of far medial loss (Fig. 4k, l).

Two weeks after injury there was 37% axon degeneration and a loss of approximately 50% of axons (Fig. 4m, n). At 4 weeks after injury, there were both fewer total axons and fewer degenerative profiles (Fig. 4m, n). Mice on the low-VitC diet did not exhibit a statistically significant loss of axons until 4 weeks post injury (Fig. 4m). However, these mice had degenerative profiles at both time points suggesting prolongation of the window of axon degeneration (Fig. 4n). Glial area was increased to the same level in the ON of low-VitC mice regardless of injury (Fig. 4o). In combination this may suggest lack of appropriate phagocytosis of degenerating axons in the low-VitC-diet group, thus explaining the higher numbers of total and degenerating axons detected in these mice as compared to those on a control diet. The total axons in the ON from the high-VitE mice was similar to that in shams at both post-injury time points (Fig. 4m). Axon degeneration in the high-VitE mice was comparable to shams at 2 weeks after injury (Fig. 4n). However, at 4 weeks after injury 10% axon degeneration was detected in the high-VitE-treated mice (Fig. 4n). Glial area remained near sham levels at both time points in the high-VitE-diet mice (Fig. 4o). There was a 58% reduction in axon transport at 2 weeks after injury, but no additional decrease at 4 weeks (Fig. 4p). Transport in the high-VitE-diet mice was not different from that of sham, despite a trend towards the lower transport (Fig. 4p).

**Fig. 5:**
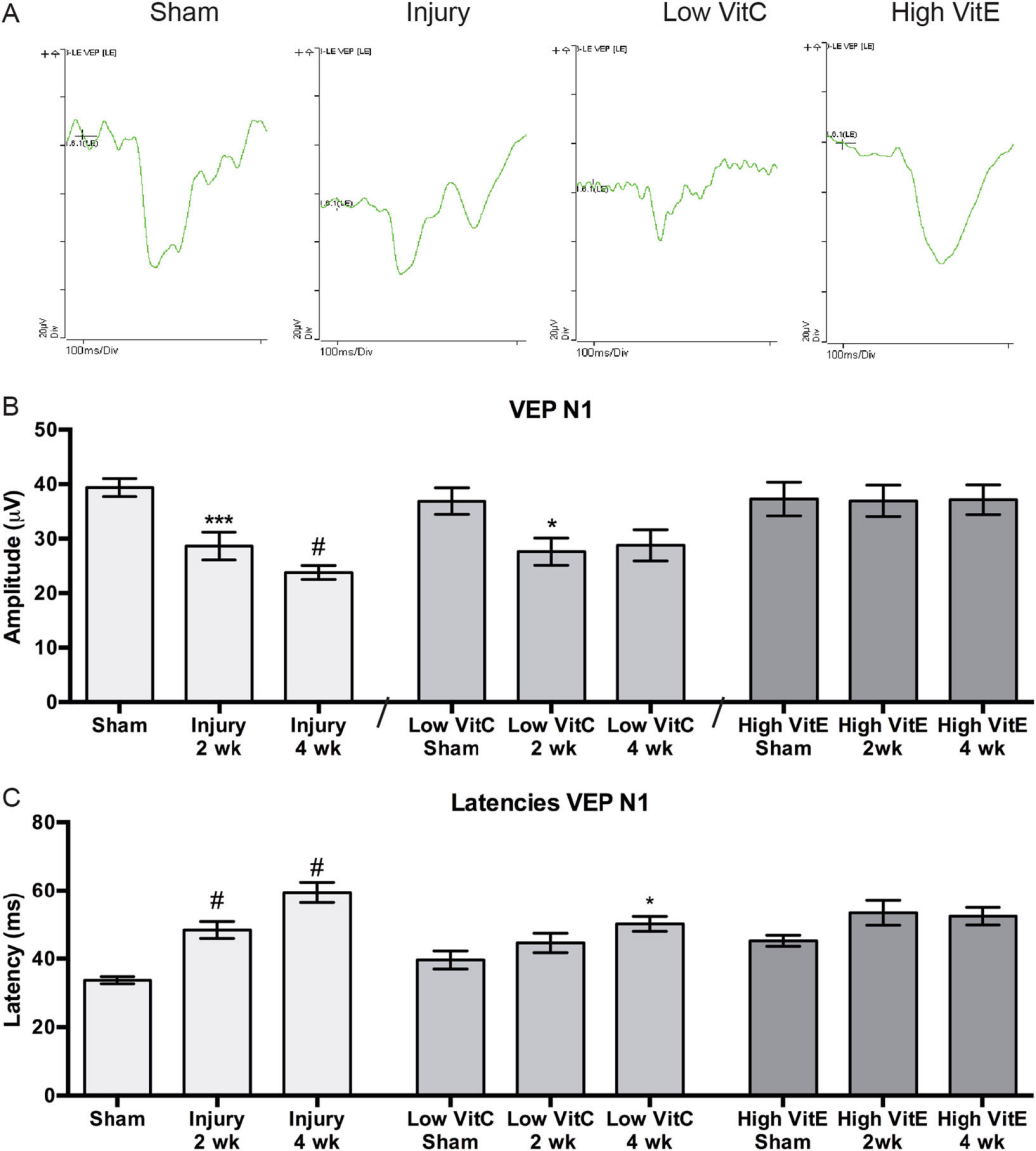
High-VitE diet prevents injury-induced decrease in the VEP N1 amplitude and latency. **a** Representative waveforms from sham, and 4 week post-injury mice on control, low-VitC, or high-VitE diets. **b** Quantification of the VEP N1 amplitude shows a decrease in both at 2 and 4 weeks after injury in mice on the control or low-VitC diets. Mice on the high-VitE diet show no decrease in amplitude at either time point. **c** Quantification of the VEP N1 latency shows increased latency that is statistically significant at 1 month after blast in both control and low-VitC-diet groups. There was no change in latency after blast in the high-VitE group. A total of 23 sham, 24 2-week-injury, 19 4-week-injury, 21 high-VitE sham, 10 high-VitE 2-week-injury, and 19 4-week-injury mice were used. *p < 0.05, ***p < 0.001, ^#^*p* < 0.0001

The visual evoked potential (VEP) waveform was diminished at 4 weeks after injury in control and low-VitC-diet mice but not in high-VitE-diet mice (Fig. 5a). The VEP N1 amplitude decreased by 27% and 40% at 2 and 4 weeks after injury, respectively, as compared to shams (Fig. 5b). It was also decreased by 37% and 49% at 2 and 4 weeks after injury, respectively, in the low-VitC-diet mice (Fig. 5b). In contrast, the amplitude in the high-VitE-diet mice was similar to shams (Fig. 5b). The VEP N1 latency was increased after injury in mice on normal or low-VitC diet (Fig. 5c). In contrast, the latency was comparable in all high-VitE-diet groups (Fig. 5c).

### **Ketogenic diet (KD) prevents the injury-induced increases in IL-1α and superoxide, but not IL-1β, and preserves the optic nerve and vision after repeat blast injury**

**Fig. 6:**
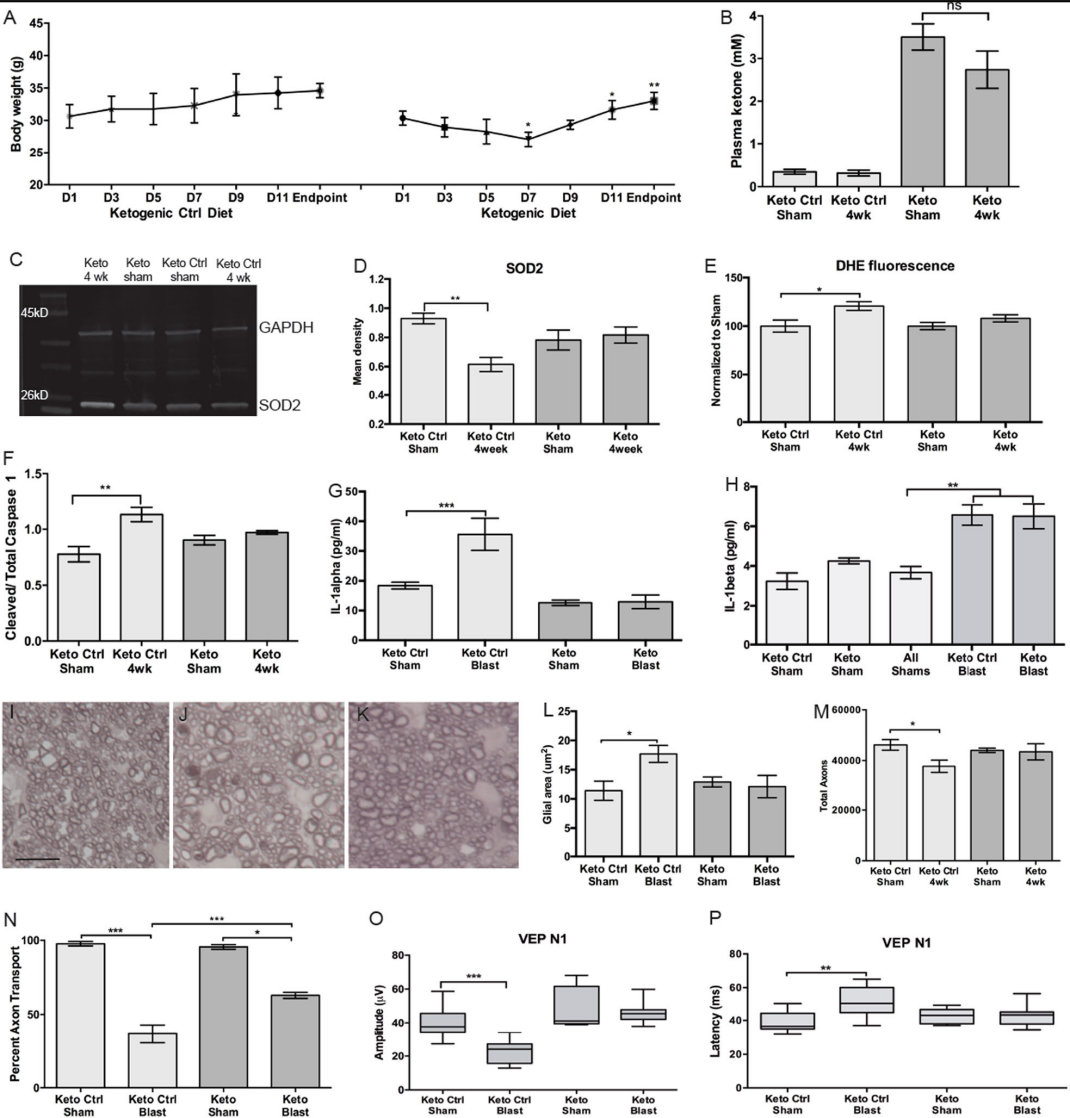
KD prevents increase in IL-1α and superoxide, but not IL-1β, and preserves axons and VEP. **a** Average mouse weight over duration of the study. Mice on the KD lost weight over the course of the first week, but regained this weight over time. Both diet groups had comparable weight at the beginning and end of the study. **b** Quantification of ketone body levels in serum of mice fed a KD or KCD. The KD increased ketone plasma levels regardless of blast exposure. **c** Representative western blot of SOD2. **d** Quantification of SOD2 levels in retinas from sham or post-blast mice fed control (KCD) or KD (Keto) diet. The KD prevented the blast-induced decrease in SOD2. **e** Quantification of DHE fluorescence in the retinas of mice on the KD or KCD. The KD prevented the increase in superoxide levels. **f** Quantification of cleaved to total caspase-1 in retinas from sham or post-blast mice fed a KCD or KD. The KD prevented activation of caspase-1. **g** Quantification of IL-1α levels in retinas from sham or post-blast mice fed a KD or KCD. The KD prevented the blast-induced increase in IL-1α. **h** Quantification of IL-1β levels in retinas from sham or post-blast mice fed a KD or KCD. Levels were increased similarly in both groups. **i**-**k** Brightfield micrographs of representative optic nerve cross-sections from KCD sham (**i**), KCD 4 weeks post-blast (**j**), and KD 4 weeks post-blast (**k**) mice. **l** Quantification of ON cross-sectional glial area in all groups showing an increase in glial area only in the KCD group. **m** Quantification of total axons in all groups showing a decrease only in the KCD group. **n** Quantification of CTB fluorescence in the superior colliculus (percent axon transport) in mice from all groups showing a decreased loss in transport in the KD group. **o**, **p** Quantification of VEP N1 amplitude (**o**) and latency (**p**) in sham and post-blast mice on all diets showing preservation of waveforms in the KD group. A total of 5 samples were used for the biochemistry and axon histology. A total of 8 KCD sham mice, 10 KCD blast mice, 5 KD sham mice, and 11 KD blast mice were used for the VEPs. *p < 0.05, **p < 0.01, ***p < 0.001

Mice fed the KD lost weight during the first week but regained overtime, and ultimately weighed the same as the ketogenic control-diet (KCD) mice (Fig. 6a). In order to confirm that the KD induced ketosis, we measured ketone body levels in the blood (Fig. 6b). Ketone bodies were increased in mice on the KD as compared to the KCD at the end of the study (Fig. 6b). Glucose levels were similar between groups (data not shown). None of the above parameters were affected by injury (Fig. 6a, b).

We measured SOD2 and superoxide levels in these mice (Fig. 6c-e). SOD2 was decreased in the retinas of mice on the KCD after blast (Fig. 6d). The KD prevented the decrease in SOD2 levels (Fig. 6d). Similarly, superoxide levels were increased in the KCD, but not KD, mice (Fig. 6e). Further, mice on the KCD exhibited increased cleaved:total caspase-1, IL-1α, and IL-1β at 4 weeks after blast (Fig. 6f–h), consistent with our results in mice on normal chow (Fig. 1). In contrast, the KD prevented the increase in cleaved:total caspase-1 and IL-1α (Fig. 6f, g). However, it did not prevent the increase in IL-1β (Fig. 6h).

The ON of 4 week post-blast mice on the KD had fewer degenerating profiles and a more normal glial phenotype than KCD mice (Fig. 6i-k). The glial area was increased post blast in the KCD ON, but not in the KD ON (Fig. 6l). Similarly, total axons were decreased in KCD mice after blast as compared to shams (Fig. 6m). Total axons were similar in KD and shams at 4 weeks post-blast (Fig. 6m). Surprisingly, axon transport was decreased in both groups after blast, although there was partial protection by the KD (Fig. 6n).

The VEP N1 amplitude decreased by 44% in the mice on the KCD as compared to its sham group (Fig. 6o). In contrast, there was no difference in the N1 amplitude of KD and sham mice (Fig. 6o). The N1 latency was increased in KCD, but not in KD mice (Fig. 6p).

## Discussion

In this study we show that repeat exposure to a non-injurious over-pressure air-blast causes greater damage than a single, higher-pressure blast. Further, ROS occurs prior to and is responsible for priming and activation of the inflammasome pathway in the retina after injury. In addition, both pathways contribute to trauma-induced secondary axon degeneration and vision loss.

Detection of greater damage after repeat exposure to a lower-level blast suggests that an initial non-injurious insult causes the ON to become susceptible to an additional injury. In our paradigm, the eye was exposed to two blasts at an interval of 0.5 s for 3 days. Future studies are needed to determine the relative importance of the blast number and the inter-blast interval. Interestingly, repeat injury appears to magnify the same molecular events rather than activating different pathways.

Injured mice had increased levels of superoxide and decreased levels of SOD2 as compared to shams, suggesting that ocular trauma causes mitochondrial dysfunction. These results complement the increase in peroxynitrite we previously detected^8^. The high-VitE diet prevented the injury-induced decrease in SOD2 and increase in superoxide. These results are similar to those reported in a rat TBI model^20^. In turn, the decrease in ROS prevented the increase in IL-1β, IL-18, and cleaved caspase-1. Thus, trauma-induced increases in ROS occur prior to and are causative for the increase in IL-1α levels, and inflammasome priming and activation. Similarly, in a glaucoma model, treatment with an antioxidant prevented inflammation and preserved the RGCs^21^. Surprisingly, although we did not detect axon degeneration at 2 weeks after injury in the high-VitE-diet mice — the peak of axon degeneration in control-diet mice — we did detect axon degeneration at 1 month after injury. Thus, the high-VitE diet blunted and delayed, but did not completely block all secondary axon degeneration. Future studies are needed to determine if axon degeneration continues past 1 month. It is possible that the axon degeneration detected at 1 month is due to ROS that is not addressed by the lipophilic VitE. This could include ROS such as hydrogen peroxide and peroxynitrite. Although the mice were also given high levels of VitC, the VitC was consumed to recycle VitE and thus probably did not have a substantial effect on directly combating injury-induced ROS. Since VitE blocked the increases in inflammasome-related proteins, the delayed axon degeneration is likely independent of that pathway.

The low-VitC diet is considered representative of partial depletion in the population that would still be considered within a clinically healthy range, i.e. not scorbutic, but is below the saturation of the VitC transporters. This suboptimal level of VitC resulted in a higher basal level of the IL-1 family pro-inflammatory cytokines and glial hypertrophy, suggesting that VitC plays an important role in the normal health and physiology of ON astrocytes. It also suggests that consuming below-saturating levels of VitC may prime the CNS for an inflammatory event, resulting in a faster and more robust response to injury.

Glial health is intrinsically linked to neuronal health, especially in the context of metabolism. To maintain normal function, neurons rely upon glia to produce lactate and pyruvate for oxidative phosphorylation^22–24^. This metabolic collaboration between neurons and astrocytes is exceptionally important during periods of stress, injury, or neurodegenerative disease, each of which tax normal energy utilization^25,26^. During the early stages of pathology, astrocytes remodel, and utilize energy reserves, likely preserving axonal function through chronic stressors^27–29^. In both control mice and mice with low VitC, blast generates a glial response similar to that seen in late stages of neurodegenerative disease, where glia become hypertrophic^30^. In contrast, the ON from high-VitE mice demonstrated both glial and axonal morphology visually indistinguishable from that of sham mice (Fig. 4). This suggests that glial hypertrophy and concomitant axonal degeneration are closely associated with ROS and the sterile inflammation pathway.

A KD has been reported to be protective in other neurodegenerative conditions, including a model of TBI^31–36^. This diet affects metabolism, ROS, and inflammation, each of which could contribute to the neuroprotective effect (for a review see ref. ^37^). Our results demonstrate a partial effect on ROS and an inhibition of inflammasome activation, but not priming. Others have shown that the ketone body, β-hydroxybutyrate, can prevent both priming and activation of the NLRP3 inflammasome^38,39^. The difference in results could be dose-, NRLP subtype, and/or cell type-dependent, as both previous studies were performed in peripheral immune cells using direct delivery of different doses of β-hydroxybutyrate.

Interestingly, the KCD provided an antioxidant benefit as compared to mice on normal chow. Superoxide levels were increased 53% and SOD2 levels were decreased 72% in 4 week post-blast normal chow mice as compared to shams. In contrast, superoxide levels were increased 21% and SOD2 levels were decreased 34% in mice on a KCD as compared to shams at 4 weeks post-blast. Despite this, the KCD mice exhibited increases in the inflammasome components comparable to mice on a normal chow. Interestingly, even though the KCD did not prevent activation of the inflammasome, the decrease in ROS resulted in better axon preservation as compared to mice on normal chow: 18% versus 51% axon loss, respectively. Therefore, ROS contribute to axon degeneration independently of activation of the inflammasome.

The KD blocked ROS and inflammasome activation and protected the ON and vision. However, it did not prevent priming of the inflammasome as evidenced by the increase in IL-1β levels. Thus, the antioxidant effect of the KD may not be as robust as VitE, resulting in the presence of ROS other than superoxide, resulting in priming, but not activation of the inflammasome. Two strong candidates are nitric oxide and hydrogen peroxide since we previously detected peroxynitrite^8^, and evidence of the Fenton reaction upon increased iron delivery after trauma^40^.

Although we previously detected caspase-1 and nitrotyrosine in a small area of inner retina at 3 days after blast, we did not detect increases in cleaved caspase-1 or IL-1β in this study until 4 weeks after blast. This could be due to the insensitivity of whole-retina Western blot and ELISA assays. It is unclear why the increase in IL-18 was transient.

In summary, the increase in ROS contributes to trauma-induced axon degeneration directly and through activation of the inflammasome pathway. Our data support clinical findings showing a correlation between low VitC levels and neurobehavioral scores in TBI patients^41^. The results of the dietary interventions demonstrate that blast-induced ROS occurs prior to increase in IL-1α and activation of the inflammasome. Based on the order of events, a combination treatment targeting ROS and the inflammasome pathway will likely be most effective as a post-injury treatment.

Although the increase in IL-1α is secondary to ROS, it could still be the primary injury signal. We intend to explore this in future studies. Future studies should also assess the therapeutic efficacy of intervening with ROS or the inflammation pathway after injury. Future investigations could also explore the death of the RGC and utilize alternative measures of visual function such as the optokinetic reflex. Finally, future studies should explore which cells, or cell types, drive the ROS and inflammasome response after injury.

## Materials and methods

### Mice

C57Bl/6 mice were purchased from Jackson Laboratories (Bar Harbor, ME). Gulonolactone oxidase knock-out (Gulo^–/–^) mice were provided by the co-author, Fiona Harrison^14^. Low-VitC mice were Gulo^–/–^ mice given deionized water containing 0.03 g/L ascorbic acid and 20 μl/L EDTA for 1 month prior to injury and for the duration of the experiment. The EDTA is included to improve the stability of VitC in the water^14^. The high-VitE mice were fed diet D04101102 (Research Diets, Inc, New Brunswick, NJ) for 1 month prior to injury and maintained on the diet for the duration of the experiment. Based on average mouse daily food intake, it is estimated that the mice consumed 50 mg/kg body weight/day VitE and 100 mg/kg VitC. The KD (TD.150843, Teklad) and KCD (TD.150844; Teklad) were purchased from Envigo (Indianapolis, IN). Mice for the ketogenic study were put on the diets 2 weeks prior to injury and maintained on the diets for the duration of the experiment. All procedures were performed in accordance with the VUMC Institutional Animal Care and Use Committee-approved protocol and Association for Research in Vision and Ophthalmology guidelines. All mice were used at 3–4 months of age. Only male mice were used for this study. Mice were perfused with PBS and 4% paraformaldehyde at collection.

### Ocular trauma

Eye injury was induced as previously described^8^. Briefly, mice were anesthetized with 2.5% isofluorane and secured into a padded housing chamber. The housing chamber was placed inside of a pipe. The left eye of the mouse was positioned against the hole in the pipe, which was aligned with the barrel of the paintball marker. All experiments were performed in the morning. Mice were either exposed to a single 26 psi blast of air (single), or to two back-to-back blasts of 15 psi air that were then repeated daily for 3 days (repeat). Sham mice were anesthetized and placed in the holder with a barrier between the barrel and the eye such that mice were exposed to the sound but not the pressure from the air-blast. Sham mice were collected at the same time-points as the blast-exposed mice. Mice were provided gel recovery food (Clear H_2_O, Portland, ME) for the first 3 days post-injury.

### Visual evoked potentials

Mice were dark-adapted overnight, dilated with tropicamide for 10 min, and anesthetized with 20 mg/kg ketamine/ 8 mg/kg xylazine/ 8 mg/kg urethane. Mice were placed on the heated surface of the ERG system to maintain body temperature. Corneal electrodes with integrated stimulators were placed on the lubricated corneas using the Celeris system (Diagnosys LLC, Lowell, MA). Subdermal platinum electrodes were placed in the snout and back of the head at the location of the visual cortex. Each eye was separately exposed to 50 flashes of 1 Hz, 0.5 cds/m^2^ white light through the corneal stimulators and responses in the visual cortex were recorded for 300 ms at 2000 Hz.

### **In vivo imaging**

Mice were anesthetized with 2.5% isofluorane and intravitreally injected with 1 μl of dihydroethidium (DHE; ThermoFisher Scientific, Waltham, MA) in phosphate-buffered saline (PBS) using a 30-gauge Hamilton syringe. Just prior to imaging, mice were anesthetized with ketamine/xylazine and eyes were dilated with 1% tropicamide. Thirty minutes after DHE injection, fluorescence was imaged on a Micron IV retinal imaging microscope (Phoenix Research Labs, Pleasanton, CA) using an FF02-475/50 nm excitation filter (Semrock, Inc. Rochester, NY) and ET620/60X emission filter (Chroma Technology Corp., Bellows Falls, VT). Using ImageJ, the average intensity of the fluorescence throughout the retina was quantified. Data were analyzed in Graphpad Prism. Mice collected 4 weeks after injury were imaged just prior to collection. Mice collected 2 weeks after injury were imaged at 1 week after injury.

### Axon transport

Axon transport assessment was performed as previously published^42–45^. Seventy two hours prior to perfusion, mice were anesthetized with 2.5% isofluorane and 1 μl of cholera toxin subunit B (CTB) conjugated to Alexa-Fluor 488 (ThermoFisher Scientific, Waltham, MA) was intravitreally injected using a 30-g Hamilton syringe. After collection, the cortex was dissected away and the brain was cryopreserved in 30% sucrose in PBS overnight. Fifty-micron thick coronal mid-brain sections were collected on a Leica SM 2000R freezing slide microtome (Leica Biosystems, Buffalo Grove, IL). Alternating sections through the superior colliculus were imaged on an Olympus AX70 microscope. Fluorescence intensity was quantified using a custom ImagePro macro. After normalizing to background, CTB intensity was calculated to construct a retinotopic map of intact anterograde transport across the superior colliculus. Topography density was found by tracing the region of the retinotopic map with intensity ≥70% of the maximum CTB signal for that tissue. Volume density is defined as the volume of superior colliculus with intact transport as a percent of total tissue volume.

### ON histology

Small pieces of proximal ON were post-fixed in glutaraldehyde and embedded in Resin 812 and Araldite 502 (cat 14900 and 10900, respectively; Electron Microscopy Sciences, Hatfield, PA) according to previously published protocol^8,40,42,46^. One-micron thick sections were collected on a Leica EM-UC7 microtome and stained with 1% paraphenylenediamine (ppd) and 1% toluidine blue. Cross-sections were imaged on a Nikon Eclipse Ni-E microscope (Nikon Instruments Inc., Melville, NY) using a 100x oil immersion objective. Total and degenerating axons were quantified using Image J. A grid was used to count 20% of the optic nerve cross-sectional surface area to avoid bias. Total and degenerating axons were counted. A series of Matlab (MathWorks, Natick, MA) routines was used to quantify the glial area as a fraction of the total cross-sectional area of the nerve^47^. Briefly, these routines detect boundaries in contrast-enhanced ON cross-sections and utilize a series of filters to exclude non-glial elements. The routine outputs a binary image with glia highlighted in white and the remainder of the nerve in black. Glial area is summed and divided by total nerve area to quantify the percent glial coverage.

### Blood glucose

A drop of blood was placed onto an Accu-chek test strip following the manufacturer's directions (Roche Diabetes Care, Inc., Indianapolis, IN).

### Ketone assay

Serum was collected in Z tubes (cat. 450470; Greiner Bio-one, Kremsmunster, Austria). Approximately 5–10 μl of serum was used in a ketone body assay kit, which was used according to the manufacturer's directions (Sigma Aldrich, St. Louis, MO).

### ELISA

A multiplex cytokine ELISA was used according to the manufacturer's directions (Millipore, Burlington, MA). An IL-18 ELISA was used according to the manufacturer's directions (Abcam, Cambridge, MA). Inter and intra-variability were previously demonstrated to be low for these kits^48^.

### Western blot

Single retinas were homogenized and sonicated in lysis buffer and centrifuged. Sample buffer was added to the supernatant just prior to use. Known amounts of protein (10 to 20 μg) or protein ladder were loaded into each well of an SDS-polyacrylamide gel. The Bio-Rad mini-trans blot cell system and mini protean pre-cast gells at 4–20% were used (Hercules, CA). Loading controls included GAPDH (1:1000; ab9485, Abcam, Cambridge, MA) or HSP60 (1:1000; ab45134, Abcam). The protein was transferred onto nitrocellulose using the Bio-Rad trans blot turbo transfer system (Hercules, CA) and alkaline phosphatase was used for band detection. Band density was quantified by scanning the blot using an EPSON scanner and Adobe Photoshop to convert to grayscale and invert the image. Each band was selected with the same frame and set measurements were used to obtain the greay mean value for each. Antibodies used were anti-caspase 1 (1:1000; ab108362, Abcam), anti-SOD2 (1:1000; ab13533; Abcam), anti-TXNIP (1:1000; 14715, Cell Signaling Technology, Danvers, MA), anti-NLRP1 (1:1000; 4990, Cell Signaling Technology), and anti-NLRP3 (1:1000; 15101, Cell Signaling Technology).

### HPLC

Contralateral retinas were used for HPLC quantification of VitE and VitC levels according to previously published protocol^14^. Briefly, ascorbic acid concentrations were measured by ion pair HPLC using a Dionex Coulochem III Electrochemical detector with Ultimate 3000 quaternary analytical pump and Chromeleon software, with a Waters RadialPak C-18 column. The mobile phase was 30% methanol, 61% de-ionized water, and 8% 1 M sodium acetate stock, and 1% 100 mM tetrapentyl-ammonium bromide was used as the ion pair reagent^14^. Retinal ascorbic acid was extracted in 200 ml per retina 25% (w/v) aqueous metaphosphoric acid and 100 mM sodium phosphate buffer containing 5 mM EDTA (pH 8.0), mixed together in a ratio of 2:7. Samples were centrifuged and aliquots of the clear supernatant were taken for assay of ascorbic acid following appropriate dilution with de-ionized water. The remaining pellet was used to establish protein levels using BCA assay.

### Experimental design and statistical analysis

Sham mice were used as controls for all studies. These mice were anesthetized and placed in the holder, but the blast air-wave was blocked. All Western blot data were normalized to loading controls. Data were analyzed using GraphPad Prism6 (La Jolla, CA). Multiple groups were compared using a one-way ANOVA and the Tukey post-hoc test.
